# Defect Engineering in Atomic-Layer-Deposited Cerium Oxide

**DOI:** 10.1021/acsami.5c22734

**Published:** 2026-03-16

**Authors:** Rudi Tschammer, Marcel Schmickler, Yuliia Kosto, Karsten Henkel, Parmish Kaur, Anjana Devi, Carlos Morales, Jan Ingo Flege

**Affiliations:** † Applied Physics and Semiconductor Spectroscopy, 38871 BTU Cottbus-Senftenberg, Cottbus 03046, Germany; ‡ 9142 Leibniz Institute for Solid State and Materials Research, Dresden 01069, Germany; § Faculty of Mathematics and Physics, Department of Surface and Plasma Science, Charles University, V Holešovičkách 2, Prague 8 180 00, Czech Republic; ∥ Inorganic Materials Chemistry, Ruhr University Bochum, Bochum 44801, Germany; ⊥ Chair of Materials Chemistry, TU Dresden, Dresden 01069, Germany; # Fraunhofer Institute for Microelectronic Circuits and Systems (IMS), Duisburg 47057, Germany

**Keywords:** cerium oxide, atomic layer deposition, X-ray photoelectron spectroscopy, inelastic peak shape analysis, defect engineering, ultrathin films

## Abstract

This study explores the role of atomic layer deposition (ALD) as an enabling technique for the defect engineering of catalytically active ultrathin deposits. In particular, we demonstrate the feasibility of tuning the O/Ce ratio in thermal ALD-based cerium oxide layers grown on silicon-based or alumina substrates by using the organometallic precursor tris­(N, N’-diisopropyl-2-dimethylamido-guanidinato)­cerium­(III) ([Ce­(dpdmg)_3_]) with H_2_O, O_2_, or O_3_ as coreactants. As revealed by *in situ* X-ray photoelectron spectroscopy (XPS), the Ce^3+^ concentration, i.e., the concentration of oxygen vacancies, depends strongly on three factors: the type of oxygen source, the chosen substrate, and the film thickness. The fixation of Ce^3+^ states during the early stages of growth is primarily determined by interface formation and the appearance of silicate and aluminate species, along with changes in morphology and surface-to-volume ratio. For thicker deposits (>5 nm), the intrinsic oxygen vacancies are coreactant-dependent. Furthermore, the chosen oxygen source also influences the morphology of ultrathin deposits, enabling potential surface functionalization with ceria nanoislands of varying composition and size. We point to a likely connection between this chemical and morphological tuning and changes in the ALD reaction pathway. The evolution of different nitrogen and carbon species depends on the oxygen source and the number of ALD cycles, indicating a shift in the ALD reaction mechanism from ligand exchange using H_2_O to ligand combustion for O_3_. The comprehensive investigation of these growth parameters is crucial for tailoring film properties via precise defect engineering.

## Graphical abstract

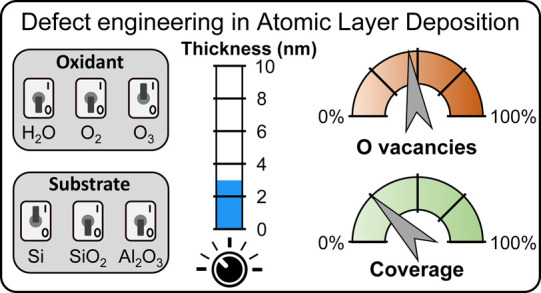

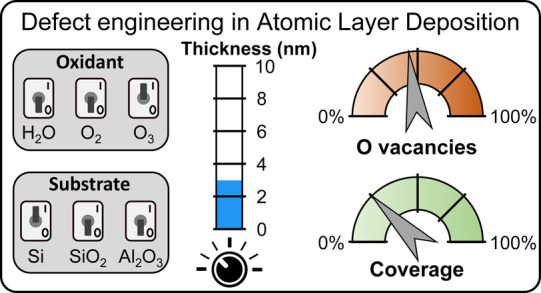

## Introduction

Given the increasingly notable effects of climate change, there is an urgent need to reduce and mitigate anthropogenic CO_2_ emissions.^1^ The transformation of the current fossil-fuel-based energy system and transportation sectors toward sustainable energy sources represents a critical turning point. In this transition, generating hydrogen (H_2_) from renewable energy sources and closing the CO_2_ cycle are two promising and complementary solutions, particularly for applications where electrification is not feasible.^2–4^ The synthesis and utilization of synthetic energy carriers—whether H_2_, hydrocarbons, or alcohols—require highly efficient and selective catalysts, as well as the development of a new generation of sensing materials capable of operating at ambient conditions and readily integrable in mass devices. Importantly, high performance in catalysts and sensing active films is usually achieved through scarce noble metals and rare-earth compounds,^5^ which compromises their competitiveness against current fossil fuels and extensive adoption. Despite the constant efforts to substitute these materials, their superior properties make them irreplaceable in many fields and applications. Current attempts focus on finding their optimal minimum usage while enhancing their properties to overcome cost-performance challenges.

In this context, atomic layer deposition (ALD) emerges as an enabling technique, offering exceptional control over deposit thickness and conformality, along with high flexibility in material selection and composition. This precise controllability, combined with the potential for industrial scalability, originates from its self-limiting chemistry: sequential pulses of precursors and coreactants that can react only with available surface sites within each subcycle.^6^ For instance, ALD has attracted considerable interest for the synthesis of heterogeneous and electrochemical catalysts^7–9^ as well as sensing active films,^10^ including both mixed compounds^11^ and elemental deposits such as nanometric noble metal clusters.^12–14^ In stark contrast to highly ordered epitaxial films typically investigated for advanced applications,^15^ low-temperature-grown ALD films are often structurally disordered, defect-rich, and nonstoichiometric, rendering their structural analysis experimentally and theoretically more complex. However, the increased complexity of ALD-prepared films may give rise to new defect chemistries that modify and potentially enhance their material properties compared to their well-ordered counterparts. Moreover, changes in the ALD reaction mechanism between the heterodeposition (initial precursor-substrate interaction) and homodeposition (bulk-like growth on material deposited in previous cycles) regimes, along with interactions between the film and substrate materials, frequently result in complex interfaces that also influence the physicochemical properties of active layers.^16,17^ As amorphous materials have been shown to improve the catalytic performance in some casess,^18–20^ the intrinsic nature of ALD to create nearly amorphous, highly defective films may be leveraged as a tool for controlled defect engineering. Consequently, precise adjustment of ALD growth parameters—including temperature, choice of precursor and coreactant, thickness, and substrate selection—enables fine-tuning of both the type and quantity of structural and chemical defects.

This direct link between process parameters and the concentration of specific defects is referred to as “defect engineering”. On the one hand, structural defects, i.e., disordered structural motifs present in amorphous deposits that deviate from their crystalline counterparts, are primarily influenced by growth temperature; however, their evolution is not discussed in this manuscript, as deposition temperature has not been varied. On the other hand, chemical defects refer to deviations from the ideal composition, either through oxygen vacancies in oxides or through the incorporation of foreign atoms, typically from precursor residues. These defects influence the sample’s surface chemistry and can be monitored via surface-sensitive techniques, such as X-ray photoelectron spectroscopy (XPS).

Here, we have investigated defect engineering in the reducible, rare-earth metal oxide ceria (CeO_*x*_ 1.5 < *x* < 2). Its reversible transition between its Ce^3+^ and Ce^4+^ cationic states depends on environmental conditions, rendering ceria a promising candidate for sensing applications^21–25^ and establishing it as a well-known catalyst for various catalytic reactions, including the conversion of carbon dioxide (CO_2_) to methanol,^26^ oxidation of carbon monoxide (CO),^27^ and the processing of volatile organic compounds (VOCs).^28^ Besides, the presence of Ce^3+^ defects has proven beneficial for gas-sensing applications, e.g., acetone^29^ and NO_2_,^30^ and for catalytic performance in CO oxidation^31^ and the water–gas shift reaction.^32^ In these examples, Ce^3+^ surface species were promoted via postsynthesis treatments, including H_2_ annealing^29^ and chemical treatment with ascorbic acid,^30^ morphology-selective synthesis,^31^ or by Sm-doping.^32^

In this work, we demonstrate control over the Ce^3+^ defect concentration by selecting appropriate ALD process parameters. Compared with deposits of higher crystalline order, the elevated defect concentration—both structural and chemical—in ALD-based cerium oxide layers enhances the formation, diffusion, and recovery of oxygen vacancies. This hypothesis was recently tested by ultrathin CeO_*x*_ layers prepared by ALD using the commercial [Ce­(thd)_4_] precursor and O_3_, which exhibited significant reduction through heterolytic H_2_ activation at room temperature during exposure to H_2_/O_2_ mixtures of varying concentrations.^16^ This superior behavior, relative to physical vapor deposited (PVD) films, was attributed to the presence of defects in the cerium oxide film originating from the ALD process, including low crystalline order and oxygen-deficient composition of as-grown deposits.^33^ As the next step, effectively tuning the physicochemical properties of ceria deposits by defect engineering using ALD requires a direct relationship between the film’s growth conditions and intrinsic properties, such as crystallinity (via temperature control) or Ce/O ratio (via coreactant choice).

In this context, the chemistry of the metal–organic precursor is key. Several investigations focused on CeO_*x*_ ALD using different precursors have been published (see Table 1). While ceria alkoxides, such as tetrakis­[1-(methoxy-κO)-2-methyl-2-propanolato]­cerium ([Ce­(mmp)_4_]),^39–42^ exhibit high reactivity and moderate growth rates, their low volatility and tendency toward oligomerization necessitate liquid injection systems, complicating their application. The commonly used β-diketonate tetrakis­(2,2,6,6-tetramethyl-3,5-heptanedionato)­cerium, [Ce­(thd)_4_],^33–38^ exhibits low reactivity toward mild coreactants and low growth rates, and typically requires high growth temperatures above 200 °C. Similar concerns may be raised for the established tris­(isopropylcyclopentadienyl)­cerium precursor, [Ce­(iPrCp)_3_].^47–49^ The heteroleptic cerium precursor bis­(isopropylcyclopentadienyl)­(N,N’-diisopropylacetamidinato)­cerium, [Ce­(iPrCp)_2_(iPr-AMD)] ^44–46^ addresses these challenges by exhibiting high reactivity toward H_2_O and growth rates around 1.5 Å just below 200 °C, although the precursor synthesis may be a delicate process with low yields.^50^ Building on these results, the homoleptic tris-amidinate precursor tris­(N,N’-diisopropylacetamidinato)­cerium, [Ce­(N-iPr-AMD)_3_][Bibr ref43] has been developed and shows an exceptional growth rate of 2.8 Å, but suffers from high growth temperatures.

**Table 1:** Overview of Reported Precursors for Cerium Oxide ALD, Including the ALD Temperature Window, Growth per Cycle, Coreactants, and Associated Challenges^a^

Precursor	ALD temperature window	Growth per cycle	Co-reactant(s)	Challenges
Ce(thd)_4_ ^33–38^	175 °C–400 °C	0.2 Å–0.4 Å	O_3_, air^38^	Low reactivity to mild coreactants
Ce(mmp)_4_ ^39–42^	150 °C–350 °C	1 Å–1.7 Å	H_2_O	Low volatility, liquid injection necessary
Ce(N-iPr-AMD)_3_ ^43^	220 °C–255 °C	2.8 Å	O_3_	High growth temperature
Ce(iPrCp)_2_(N-iPr-AMD)^44–46^	185 °C–265 °C	1.5 Å–1.9 Å	H_2_O	Low-yield synthesis
Ce(iPrCp)_3_ ^47–49^	250 °C – 275 °C	0.2 Å–0.35 Å	O_2_ plasma, H_2_O	low growth rate, high growth temperature
Ce(dpdmg)_3_ ^50^	160 °C	2.1 Å	H_2_O	

a Ligand abbreviations are explained in the text.

In this work, we use the homoleptic tris-guanidinate precursor [Ce­(dpdmg)_3_] (dpdmg = N,N’-diisopropyl-2-dimethylamido-guanidinato) first reported by Kaur et al.,^50^ which exhibits high reactivity toward H_2_O, an excellent growth rate of 2.1 Å at 160 °C, and comparatively high volatility at 140 °C. Additionally, density functional theory (DFT) calculations indicated the potential use of multiple oxidants, each associated with distinct reaction pathways, that could lead to differences in the Ce/O ratio and potential residues. Building on this flexibility, we explore oxygen (O_2_) and ozone (O_3_) as alternative coreactants and demonstrate the viability of defect engineering in ultrathin (<20 nm) cerium oxide layers grown by ALD. Our findings indicate precise control over the Ce cation oxidation state, as well as the type and amount of carbon- and nitrogen-based chemical residues, by adjusting key parameters of the ceria ALD process, such as the total number of cycles (i.e., thickness) and the selected coreactant. These results are supported by *in situ* X-ray photoelectron spectroscopy measurements, an approach that enables facile determination of these properties as a function of deposit thickness via stepwise ALD without potential sample alteration from atmospheric exposure.^33^ Furthermore, we ascertain the thickness and morphology of the ALD deposits through qualitative *operando* ellipsometry and quantitative inelastic peak shape analysis (IPSA) derived from XPS measurements.

The integration of these *operando* and *in situ* characterization techniques enables a comprehensive description of the early growth stages, including interface formation, the Ce^3+^/Ce^4+^ gradient, and the evolution of chemical defects. Achieving precise control over film properties through defect engineering of ultrathin ALD deposits enables targeted enhancement of catalytic and sensing behavior while minimizing the consumption of scarce materials and maintaining compatibility with industrial processes.

## Experimental Methods

Experiments were conducted in an ultra-high vacuum (UHV) ALD-XPS cluster setup described elsewhere.^51^ Cerium oxide films were grown on silicon (100) substrates (Crystec GmbH) with a thin layer of native oxide of around 2.4 nm, as estimated by spectroscopic ellipsometry; thermally grown SiO_2_ (∼300 nm) on a silicon (100) substrate (Crystec GmbH); and ALD-Al_2_O_3_ on Si (25 nm, SENTECH Instruments GmbH).^52^ The thermal-ALD growth of cerium oxide was performed using tris­(N,N’-diisopropyl-2-dimethyl-amido-guanidinato)­cerium­(III) ([Ce­(dpdmg)_3_]) and H_2_O (spectrophotometric grade, Alfa Aesar), O_2_ (99.9999%, Air Liquide), or O_3_/O_2_ (∼7%, vol % O_3_). The ozone was generated by an OXP-30 ozone generator from Oxidation Technologies, fed with 99.9999% O_2_ (Air Liquide). Before deposition, substrates were cleaned by heating to 250 °C under UHV conditions for at least 30 min, until XPS measurements confirmed minimal carbon contamination.

The [Ce­(dpdmg)_3_] precursor was synthesized according to published procedures^50^ and kept in a stainless steel bubbler, heated to 140 °C, with N_2_ (99.9999%, Air Liquide) as the carrier gas flowing through the bubbler and pushing the precursor vapor to the reactor. The ALD reactor walls and precursor gas lines were kept at 140 °C and 120 °C, respectively. When using H_2_O as the oxidizing agent, the coreactant gas lines were heated to 90 °C, while the water was kept in a glass vial at room temperature. During the deposition process, the ALD reactor was operated in flow-type mode with N_2_ as the carrier gas. The N_2_ gas flow was set to 160 and 110 sccm for the precursor and coreactant lines, respectively, by separate Bronkhorst F-111B 200 mass-flow controllers, and the gas dose was alternately switched using pneumatic ALD valves (ALD3 series, Swagelok), controlled with a homemade LabVIEW (2020 SP1) program. The precursor half-cycle of the ALD process consisted of a [Ce­(dpdmg)_3_] pulse of 13 s, followed by a 1 s N_2_ pulse and a remaining purging step of 4 s for the precursor line, and continued by pumping the ALD reactor for 23 s. Depending on the oxidizing agent, the second half-cycle consisted of a coreactant pulse of 3.5 s for H_2_O, 11 s for O_3_, and 0.1 s, followed by 7 s of pumping before the subsequent purge step. Finally, the cycle was closed with a 3 s purge with N_2_ through the coreactant line and 60 s of reactor pumping. Between cycles, the base pressure was approximately 4·10^–2^ mbar.

*Operando* spectroscopic ellipsometry measurements were carried out using a SER 801 UV–vis ellipsometer from SENTECH Instruments GmbH within a wavelength range of 320–1000 nm. Determination of optical properties and layer thickness was performed using SpectraRay4 software. Depending on the sample composition, the optical model consisted of a silicon substrate with built-in temperature dependence, a SiO_2_ or Al_2_O_3_ layer, a CeO_2_ film (with optical properties taken from the SpectraRay4 materials database), and air. The initial thicknesses of the SiO_2_ and Al_2_O_3_ oxide layers were determined *in situ* after surface cleaning and were fixed during subsequent cerium oxide deposition.

XPS measurements were recorded *in situ* in an analysis chamber with a base pressure of 10^–10^ mbar using an Omicron EA125 hemispherical energy analyzer and nonmonochromatized Mg K_α_ radiation to avoid overlap of the Ce 3d core level and the Ce MNN Auger line. The pass energy was set to 20 eV, resulting in an overall resolution of around 1.0 eV.^51^ The initial calibration of the energy scale and resolution of the XPS system was carried out using a polycrystalline silver (Ag 3d_5/2_ at 368.2 eV) film deposited *in situ* by thermal evaporation. The sample charging was corrected considering the uˈˈˈ component (917.0 eV) of the Ce 3d region as an internal reference. Peak fitting of the Ce 3d core level was done following established methods by Romeo et al.^53^ and Kotani et al.,^54^ using Gaussian–Lorentzian (G-L) sum peaks and subtracting a blend of linear and Shirley background in CasaXPS 2.3.25. Notably, an asymmetry was introduced to the u/v and uˈˈ/vˈˈ doublets, similar to Skála and coworkers^55^ and Morales and coworkers.^16^ We used reference CeO_2_ and Ce_2_O_3_ samples grown by reactive e-beam evaporation and measured them in the same instrument to obtain appropriate constraints for our fit model in terms of peak separation, full width at half-maximum (FWHM), G-L ratio, and peak area relative to uˈˈˈ and v_0_ for CeO_2_ and Ce_2_O_3_, respectively. A table summarizing the constraints applied to the peak parameters is provided in the Supporting Information (Table S1). A rough estimate of the uncertainties inherent in the fitting model and associated with the Poisson nature of electron counting yields an uncertainty between 5 and 10% for the determined Ce^3+^ concentration. The C 1s spectra have been fitted with a simple model accounting for C–C/C–H, C–O, C=O/O–C–O, and O–C=O species following the literature.^56^ Inelastic peak shape analysis (IPSA) was performed to determine the thickness and morphology of ALD films by using the QUASES Analyze software^57^ applied to an extended Ce 3d region. The inelastic mean free path (IMFP) of Ce 3d photoelectrons in CeO_2_ has been estimated using the TPP-2M formula by Tanuma, Powell, and Penn.^58^

## Results and Discussion

### Growth-Per-Cycle and Reaction Mechanisms

We employed *operando* spectroscopic ellipsometry to determine the ALD settings—temperature and minimum pulse lengths of precursor/coreactant—that yield the maximum growth rate on Si substrates within a saturated process. An example of the step-like evolution of film thickness typical of ALD self-limiting reactions, in this case using H_2_O as the oxygen source, is depicted in Figure 1a. First, the ALD-temperature window was explored, obtaining linear deposition with an almost constant growth per cycle (GPC) of 1.5 Å from 140 to 240 °C (Figure 1b). The outliers around 220 °C–230 °C can be attributed to the semiquantitative nature of the SE evaluation, as further outlined in the following paragraphs. These results corroborate the linear deposition reported by Kaur et al.^50^ at 160 °C. Further discussion of the GPC determined via IPSA is provided below. At 130 °C, the GPC measured by ellipsometry increased to 2 Å, indicating the onset of condensation and a mixed ALD-CVD regime. Upon further reduction to 120 °C, we observe features indicative of thick film interference in spectroscopic ellipsometry, suggesting that the cerium precursor condenses on the substrate, consistent with its melting point around 130 °C.^50^ We have not explored the upper limit of the ALD process, as these conditions are outside the scope of this study; however, thermal decomposition has been observed by Kaur et al.^59^ by thermogravimetric analysis at temperatures exceeding 270 °C. Unless otherwise noted, the following experiments have been conducted at 160 °C.

**Figure 1:**
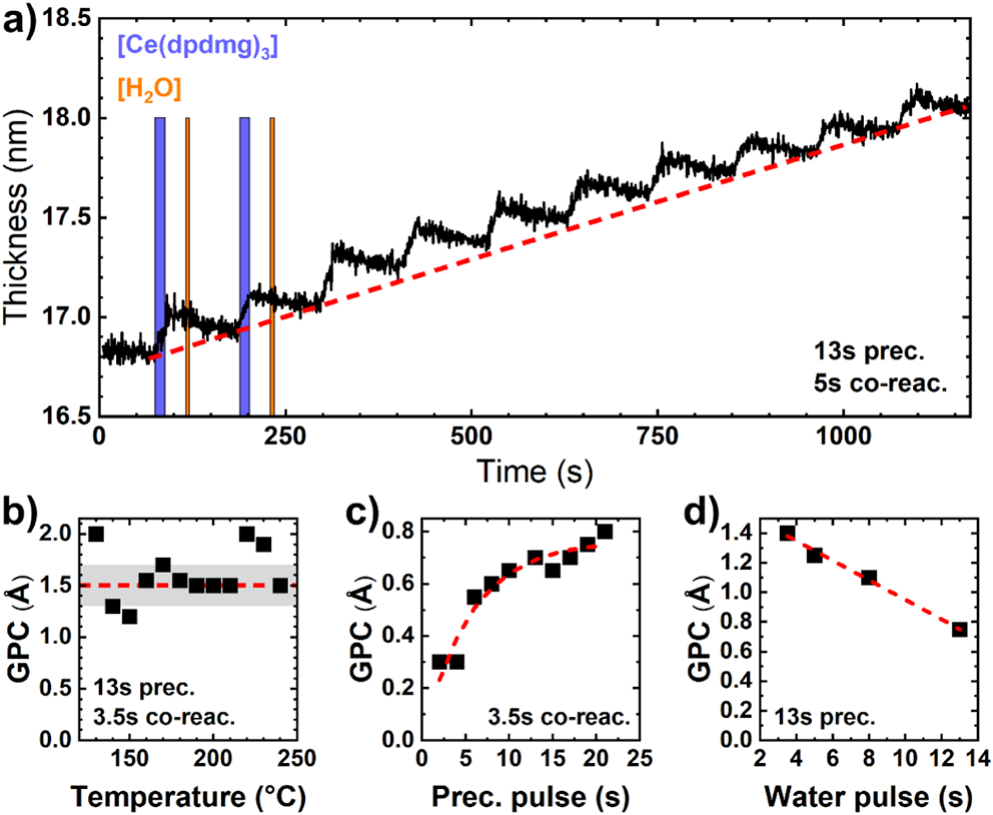
a) ALD characteristic step-like evolution of thickness as a function of time for a cerium oxide film grown via thermal ALD cycles at 160 °C, each composed of a 13 s [Ce­(dpdmg)_3_] pulse and a 5 s H_2_O pulse. Blue and orange areas indicate precursor and coreactant pulses, respectively. b) GPC of ALD-CeO_*x*_ films grown on Si substrates as a function of temperature. GPC of ALD-CeO_*x*_ films grown at 160 °C on Si substrates determined by spectroscopic ellipsometry as a function of c) precursor and d) H_2_O pulse length. Red dashed lines have been added to guide the reader’s eye.

The results of the optimization procedure for precursor and coreactant exposure when using H_2_O as an oxidizing agent are shown in Figure 1c and d. Each point represents the average GPC determined via spectroscopic ellipsometry for a set of 10 ALD cycles under the specified conditions. Precursor and water pulse lengths ranging from 2 to 23 s (Figure 1c) and 3.5 to 13 s (Figure 1d) were explored to ensure saturated exposure to each reagent. Increasing the precursor pulse length from 2 to 13 s increased the GPC from approximately 0.3 Å to 0.7 Å, indicating higher surface coverage for longer exposure times and enhanced film deposition. Extending the precursor dose time beyond 13 s does not lead to a significant increase in GPC but rather results in a gradual reduction in precursor pressure during 10 ALD cycles, consistent with insufficient precursor vapor regeneration between subsequent cycles. Accordingly, 13 s was chosen as the optimum condition for the ALD process. A water pulse length of 3.5 s, with a precursor dose of 13 s, yields a GPC of approximately 1.4 Å. Increasing the water exposure time decreases GPC, likely because water vapor is not completely regenerated between subsequent ALD cycles, as the H_2_O container is held at room temperature. Similar experiments (Figures S1 and S2 in the Supporting Information) yielded saturation dose times of 0.1 s (followed by 7 s of pumping before purging) and 11 s for O_2_ and O_3_, respectively. The short O_2_ dosing time compared to H_2_O and O_3_ can be attributed to the comparatively higher pressure behind the ALD valve, as the O_2_ gas bottle was connected directly via a pressure reducer.

To confirm the linear increase in film thickness with the number of ALD cycles, we employed inelastic peak shape analysis (IPSA) to determine the thickness and morphology of the cerium oxide films using *in situ* X-ray photoelectron spectroscopy (XPS) measurements (see Figure 2). This method, based on the work by S. Tougaard,^60,61^ estimates the thickness and provides an approximation of the morphology depending on the inelastic mean free path (IMFP) of photoelectrons in solids and the resulting change in the background of inelastically scattered electrons present in XPS spectra. Using QUASES-IMFP-TPP2M software, the estimated IMFP for Ce 3d photoelectrons excited by Mg K_α_ radiation in a CeO_2_ matrix is 8.26 Å. Although the density of cerium oxide may change when transitioning from pure Ce_2_O_3_ to pure CeO_2_, the corresponding changes in IMFP are well below 10% for a reduction from 7.2 g/cm^3^ in pure CeO_2_ to 6.2 g/cm^3^ in pure Ce_2_O_3_. Therefore, the IPSA analysis is effectively independent of changes in the cerium oxidation state and Ce/O ratio.

**Figure 2:**
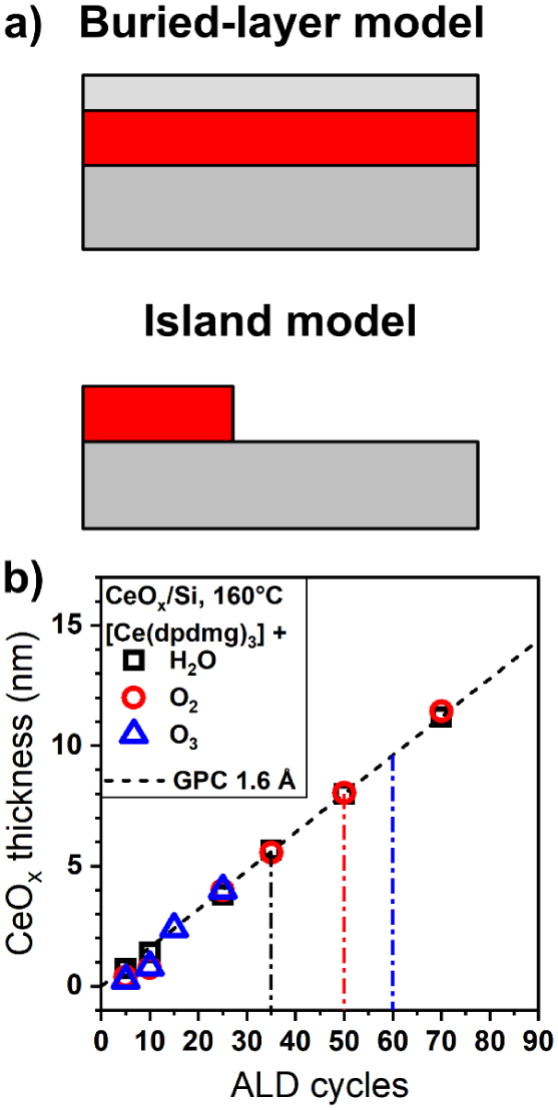
a) Schematic representations of the buried layer model (top) and island model (bottom) used for thickness determination via inelastic peak shape analysis (IPSA). The red-shaded area indicates the ALD-grown CeO_*x*_ film, whose thickness is being determined. b) Thickness of cerium oxide grown by optimized ALD processes at 160 °C on Si substrates using H_2_O (black square), O_2_ (red circle), or O_3_ (blue triangle), respectively, from IPSA. A dashed line representing a growth-per-cycle of 1.6 Å is added to guide the reader’s eye. Vertical dashed-dotted lines mark the formation of a closed CeO_*x*_ layer depending on coreactants.

Film growth via ALD usually proceeds layer-by-layer, meaning a monolayer is added during each ALD cycle (or, more precisely, a fraction of a monolayer due to steric effects and limitations on the density of nucleation sites). However, this assumption does not hold for ultrathin films, such as those studied here. Instead of a pure layer-by-layer growth mode, initial nucleation proceeds via nanoislands, with nucleation density initially determined by the number of adsorption sites on the substrate surface. These two stages, with distinct chemistry, are referred to as the homodeposition and heterodeposition regimes, respectively.^17^ With an increasing number of ALD cycles, the thickness and lateral extent of the islands increase, yielding complete coalescence of these nanoislands into a closed layer at a certain deposit thickness, thereby completing the transition from the transient heterodeposition regime to the steady homodeposition regime.

To account for this initial deviation from layer-by-layer growth, two models have been used in the IPSA analysis to determine the thickness of the cerium oxide films prepared by ALD. The first is the so-called island model (Figure 2a, bottom), in which the cerium oxide deposit is represented by one or several islands of distinct heights, while a part of the substrate remains uncovered. To avoid ambiguities, only one island has been considered in the model, allowing determination of the island height and the fraction of the substrate covered by the ALD-CeO_*x*_. The number of ALD cycles yielding a coverage of 1, i.e., the entire substrate is covered by the CeO_*x*_ film, is considered the point of coalescence. This point is indicated in Figure 2b by the vertical dash-dotted lines. While this model accurately describes the initial growth stages, during which the formation of separated nanoislands may be observed, it does not account for a potential overlayer (e.g., organic residues) that may form during the sample’s cool-down in the ALD reactor before transfer to the XPS chamber. Given this potential overestimation of deposit thickness, the main purpose of this model was to determine the film’s surface coverage.

The second model used for IPSA is the buried-layer model (Figure 2a). It assumes a stack geometry composed of three continuous layers, namely, the semi-infinite substrate, the CeO_*x*_ deposit, and a dense overlayer formed by incomplete oxidation of adsorbed organometallic molecules. The presence of such a carbon-based overlayer is consistent with *in situ* XPS measurements. Consequently, this model enables determination of the thicknesses of both the CeO_*x*_ layer and the overlayer, typically yielding overlayer thicknesses of 2–3 Å, which may be regarded as a lower limit for a dense carbon layer.^33^ While this model incorporates the layer-by-layer growth mode conventionally assumed for ALD, it does not account for partial substrate coverage, leading to a slight underestimation of thickness for noncontinuous films, i.e., in the initial growth stages. Notably, the successful use of IPSA for determining sample morphology and film thickness has been extensively validated and reviewed,^60,61^ and, more recently, specifically for ALD-based CeO_*x*_ deposits by Morales et al.,^33^ who compared their results with *ex situ* transmission electron microscopy (TEM) measurements.

Therefore, both models capture essential yet complementary aspects of the ALD process and the resulting sample morphology, as shown in Figure 2b. The island model was employed to estimate the time to reach coalescence (indicated by vertical dash-dotted lines), whereas the buried-layer model was used to estimate the average thickness for all ALD cycles, including those with incomplete substrate coverage. The thickness evolution is shown in Figure 2b for films deposited at 160 °C on Si using H_2_O, O_2_, or O_3_ as the oxygen source, respectively. Overall, it appears highly linear with a GPC of 1.6 Å for both models. This value is comparable to the GPC value of 2.1 Å reported by Kaur et al.,^50^ determined by *ex situ* X-ray reflectivity (XRR) measurements. The observed differences in the GPC values determined by IPSA and reported by XRR may be explained by the film’s partial porosity. The estimated IMFP of Ce 3d photoelectrons assumes a compact layer with a density of 7.2 g/cm^3^, significantly differing from the density of 5.0 g/cm^3^ determined by Kaur et al. from critical angle fitting.^50^ Using their density value in our analysis, i.e., by scaling our IPSA-estimated GPC by the density ratio, yields a GPC of 2.3 Å, in good agreement with Kaur’s work.

The GPC values obtained by spectroscopic ellipsometry evidently differ from those obtained by IPSA and XRR results. Both IPSA and XRR rely on the density of the cerium oxide matrix—whether atomic density of Ce cations for the former or film density in the latter—irrespective of their oxidation state; i.e., they are almost insensitive to the Ce/O ratio. In stark contrast, the analysis of spectroscopic ellipsometry data is critically influenced by differences in the optical properties of cerium oxide layers with varying composition (i.e., Ce^3+^ to Ce^4+^ ratio). However, only optical models for fully oxidized CeO_2_ bulk deposits are available. Therefore, the GPC values obtained from spectroscopic ellipsometry are useful for ALD-recipe optimization but represent a semiquantitative estimate of the true GPC due to uncertainties in the optical properties arising from varying oxide composition, and further complications related to nonidealities in the morphology of as-deposited films.

More specifically, the IPSA island model indicates that film coalescence occurs after around 35 ALD cycles when using H_2_O as the oxygen source (∼5.6 nm film thickness), while the use of O_2_ and O_3_ delays film coalescence to around 50 ALD cycles (∼8 nm film thickness) and 60 ALD cycles (∼9.6 nm thickness), respectively. This may be attributed to initial differences in nucleation site density during the respective ALD processes, with H_2_O being more efficient at regenerating reactive sites on the native oxide overlayer of the substrate. These results not only indicate the importance of the transition from the heterodeposition to the homodeposition regime,^16,17^ but also open the opportunity for surface decoration with cerium oxide nanoislands of different sizes, depending on the oxygen source used during the ALD process. In this context, nanoislands refer to ALD films before coalescence to a closed layer.

We now use the estimated GPC values in the range of 1.6–2.3 Å to better understand the ALD reaction mechanism. To this end, we have used the model developed by Puurunen,^62,63^ which relates the total number of metal atoms attached per cycle to ligand size (i.e., steric hindrance) and reactivity toward a specific surface (i.e., the availability of active sites). As our GPC value roughly corresponds to half a monolayer of cerium oxide, based on the height of an O–Ce–O trilayer,^64^ it follows that either of the two factors limits deposition. Considering that the GPC is independent of the number of ALD cycles and the intrinsic differences between native silicon oxides and cerium oxide surfaces, we can rule out the number and nature of the active sites for precursor adsorption and activation as the main factors controlling the growth. Similar to other bulky precursors, such as [Ce­(thd)_4_], a ligand exchange reaction is likely to occur during the adsorption step, while the size of the remaining ligands determines the theoretical upper limit on the GPC. Therefore, we first model the size of the guanidinato backbone of the dpdmg ligand using a simple estimation of the adsorbate size based on the data available from Kaur et al.^50^ and supplemented with the published structures of dimethylamine^65^ and isopropylamine.^66^ This simple model allows us to obtain a rough estimate of the sizes of [Ce­(dpdmg)] and [Ce­(dpdmg)_2_] adsorbates, assuming that adsorption results in the loss of two or one ligands, respectively, without resorting to more complex quantum-chemical calculations.

Figure 3 shows the results of constructing [Ce­(dpdmg)_2_] (Figure 3a,b) and [Ce­(dpdmg)] (Figure 3c,d) adsorbates. From the top view, we can estimate their size by simply placing a circle around the adsorbate, yielding equivalent radii of r_2L_ = 5.7 and r_1L_ = 4.5 Å for [Ce­(dpdmg)_2_] and [Ce­(dpdmg)], respectively (indicated by red circles in Figure 3b,d). Using Puurunen’s model, this implies estimated GPC values of 0.35 Å and 0.56 Å for both situations, respectively. As expected, the loss of two dpdmg ligands (i.e., case of [Ce­(dpdmg)]) leads to higher GPC values, but both adsorbates are still too bulky to explain the high GPC determined previously by us and Kaur et al.^50^ To explain a minimum GPC of 1.6 Å, the adsorbate size derived from the model of Puurunen corresponds to r = 2.66 Å, which is indicated by the blue circle in Figure 3d, and would imply the loss of the isopropyl-groups (C_3_H_7_) attached to the guanidinato backbone after removal of two ligands during the initial adsorption process to reduce adsorbate size sufficiently. Alternatively, a carbodiimide-deinsertion (H_7_C_3_–N=C=N–C_3_H_7_) reaction may also provide a suitable explanation, as reported for amidinate and guanidinates.^67,68^ Using the same estimations as before, a theoretical GPC of 2.34 Å is derived for an assumed adsorbate radius of 2.2 Å, which may explain the observed GPC. Both hypotheses indicate a rather complex adsorption behavior, likely common to new-generation ALD precursors with amidinate and guanidinate ligands.

**Figure 3:**
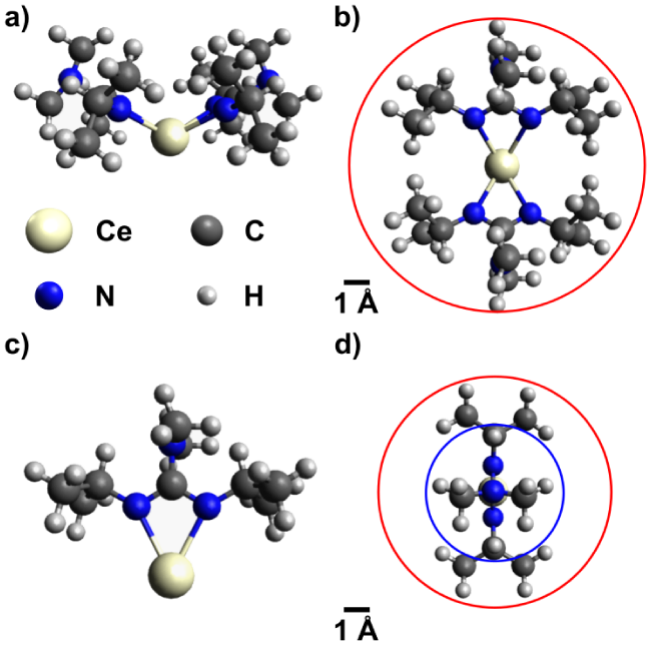
Visualization of a [Ce­(dpdmg)_2_] adsorbate in a) side view and b) top view, and of a [Ce­(dpdmg)] adsorbate in c) side view and d) top view. Red circles in b) and d) indicate the estimated adsorbate size, and the blue circle represents the adsorbate size required for an estimated GPC of 1.6 Å.

### Evolution of Cerium Oxide Cation Oxidation State

*In situ* XPS is well-suited for determining the composition and cation oxidation states of the as-deposited layers. Although *in situ* and *operando* XPS characterization of ALD deposits is becoming widespread at synchrotron radiation facilities,^69–71^ lab-based setups for *in situ* and *operando* characterization are typically limited to spectroscopic ellipsometry^45^ or infrared techniques,^72^ whereas XPS is typically relegated to an *ex situ* technique.^45,50^ This *ex situ* approach may introduce unintended changes to samples during transfer through ambient conditions, particularly for reducible oxides, where multiple oxidation states are expected to coexist.

Furthermore, multiple *in situ* studies of the physical vapor deposition of CeO_*x*_ have shown a Ce^3+^ to Ce^4+^ transition that depends on three factors: (i) interaction with the substrate;^73^ (ii) surface-to-bulk ratio,^74^ as the top surface region is more prone to reduction; and (iii) thickness, as a consequence of the previous two factors. Although high pressures and the risk of cross-contamination have prevented a surface science approach to ALD-based cerium oxide growth, similar Ce^3+^ to Ce^4+^ transitions as a function of film thickness and substrate have recently been observed for thermal-ALD using [Ce­(thd)_4_/O_3_] within a lab-based *in situ* XPS approach.^33^ Similarly, we apply this *in situ* XPS methodology to determine the Ce cation oxidation state and stoichiometry of our films, as well as the chemical residues within [Ce­(dpdmg)_3_]-based films, by analyzing how these properties depend on the chosen oxygen source and substrate.

The evolution of the Ce 3d core level of cerium oxide grown on Si using [Ce­(dpdmg)_3_] and H_2_O with increasing thickness is displayed in Figure 4a. We observe a distinct transition from a Ce^3+^-rich 0.8 nm thick ALD-CeO_*x*_ deposit to a 13.6 nm thick film containing predominantly Ce^4+^. This transition is qualitatively evident from the increase of the peak at 917.0 eV (u’’’), associated with Ce^4+^, and the concomitant decrease of the 886 eV (v’) peak, corresponding to Ce^3+^. Stabilization of the Ce^3+^ fraction occurs at 25–30% at a layer thickness of ∼5.6 nm (35 ALD cycles) (Figure 4a and b), coinciding with layer closure (Figure 4c). This supports the idea that Ce^3+^ is concentrated at the interface between the Si substrate and the ALD-deposited cerium oxide layer, as in the study mentioned above.^73^ However, complete oxidation to stoichiometric CeO_2_ is not observed when H_2_O is used as the oxygen source.

**Figure 4:**
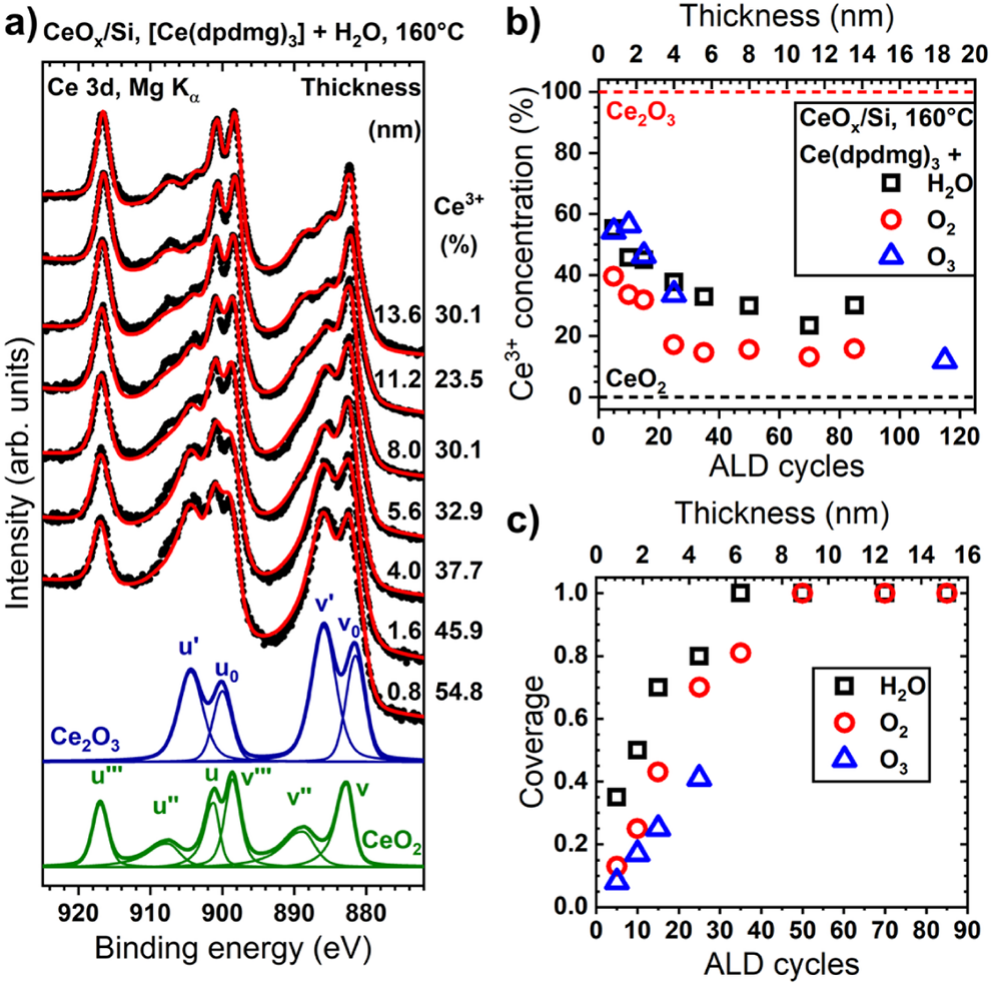
a) Evolution of the Ce 3d region as a function of the number of ALD cycles for ALD-CeO_*x*_ grown at 160 °C on Si using [Ce­(dpdmg)_3_] and H_2_O. Black dots indicate raw data, while the fit used to extract the fraction of Ce^3+^ cations is depicted in red. Green and blue curves at the bottom represent the envelope curves and individual components of pure CeO_2_ and Ce_2_O_3_ spectra from this model, respectively. Evolution of b) the concentration of Ce^3+^ and c) the coverage of ALD cerium oxide grown with H_2_O, O_2_, or O_3_ as the coreactant, respectively.

When O_2_ is used during the ALD process (Figure 4b), the Ce^3+^ fraction in the oxide film is lower than when H_2_O is employed, consistent with expectations for a coreactant with a higher oxidation potential. For thin deposits up to 1.6 nm, roughly one-third of the Ce cations are in the 3+ oxidation state, with a sharp transition near 4 nm thickness toward a “bulk” value of 15–20%, possibly indicating a more abrupt interface layer between the film and substrate. Compared to cerium oxide grown with H_2_O, using O_2_ as a coreactant results in lower coverage during the initial stages of ALD growth (Figure 4c), likely due to less efficient reactions between the [Ce­(dpdmg)_3_] adsorbates and the coreactant, causing delayed layer closure around 8 nm film thickness (∼50 ALD cycles). This demonstrates that the size of cerium oxide nanoislands can be tuned by choosing a suitable oxygen source.

The use of O_3_ results in an unexpected change in the oxidation state of the Ce cation (Figure 4b). Instead of showing a reduction similar to that observed with O_2_ and thus further decreasing Ce^3+^ levels, early growth stages exhibit a high Ce^3+^ fraction similar to that of films prepared with H_2_O. This behavior seems to contradict the high oxidation potential of O_3_. Furthermore, IPSA indicates a lower coverage than those obtained with H_2_O and O_2_ (Figure 4c), despite maintaining a growth rate of 1.6 Å/cycle. As the film thickness increases to 4 nm, the Ce^3+^ concentration in the O_3_-prepared deposits remains higher than in those prepared with O_2_, possibly due to a higher surface-to-volume ratio caused by lower coverage. Extrapolating the coverage trend suggests coalescence at about 9.6 nm (60 ALD cycles), supporting the idea that an appropriate combination of oxygen source and cycle number can finely tune the size and composition of ceria nanoislands. For thicker deposits, the ceria films become nearly fully oxidized, with Ce^3+^ concentrations dropping to around 10% when using O_3_. This demonstrates that Ce^3+^ concentrations can be controlled between approximately 50% and 10% through defect engineering, by adjusting parameters such as film thickness and the choice of oxidizing coreactant.

Figure 5a illustrates the interface formation by comparing the O 1s fitted spectra of the bare SiO_*x*_/Si substrate with 0.8 and 13.6 nm thick CeO_*x*_ layers grown with H_2_O, revealing the formation of an intermixed CeO_*x*_/SiO_*x*_/Si interface where the Ce^3+^ species are located. This analysis allows for distinguishing different oxygen-containing species within the sample: the Ce^4+^–O and Ce^3+^–O from the ALD-CeO_*x*_ film, and two substrate-related components, Si*–O* from the native SiO_2‑x_, and silicates (CeSiO_3_) at the interface.^33,73^ For samples with film thicknesses exceeding 5.6 nm, the Si components have been omitted due to the film thickness and replaced with a component corresponding to surface oxygen species such as hydroxyls and carbonates. Before the CeO_*x*_ deposition, the O 1s spectrum exhibits one main component around 532.7 eV, corresponding to Si^4+^–O bonds, with an additional shoulder at lower binding energies attributed to small traces of lower oxidation states of Si (e.g., Si^3+^) and other surface oxygen species. After 5 ALD cycles (0.8 nm), the intensity of the Si^4+^-component diminishes while new species related to Ce^3+^ and Ce^4+^ emerge, indicating cerium silicate and cerium oxide growth. After a rather thick cerium oxide layer was obtained (∼13.6 nm, 85 ALD cycles), the O 1s spectrum exhibits a distinct two-peak structure. The low-binding-energy components correspond to oxygen in the CeO_*x*_ film, while the peak at higher binding energy is attributed to oxygen in carbonaceous species and hydroxyls. Moreover, a comparison of the Ce oxidation state derived from the O 1s and Ce 3d core levels shows excellent agreement between both fits (Figure 5b). The slight tendency for the O 1s analysis to yield a higher Ce^3+^ concentration until layer closure (∼5.6 nm film thickness) is easily explained by the greater IMFP of the O 1s photoelectrons compared to the Ce 3d photoelectrons, and therefore the higher sensitivity for the film/substrate interface, where a higher concentration of Ce^3+^ is expected. For thick films, the interface is no longer accessible, and the slight underestimation of Ce^3+^ in O 1s relative to Ce 3d indicates that some Ce^3+^ cations may be located near the top surface due to its partial hydroxylation.^16,75,76^ Similar results are obtained for the O_2_ and O_3_ coreactants (cf. Figures S3 and S4), also indicating the formation of silicates derived from the Si 2s core level, as previously reported for other precursors and highly oxidizing agents, as well as for PVD-based films on Si substrates.^17,33,73^

**Figure 5:**
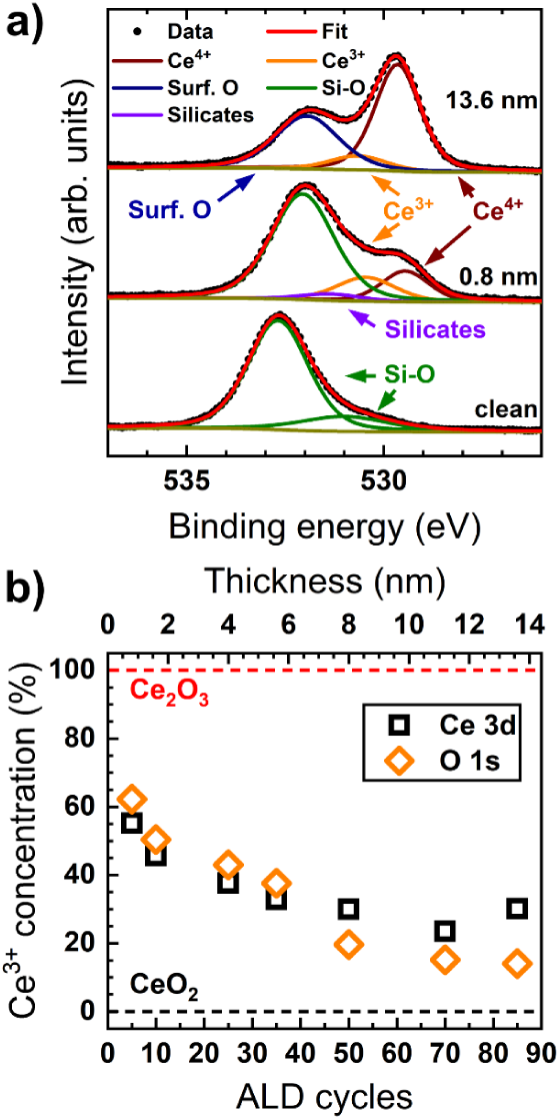
a) Comparison of the O 1s spectra of the Si substrate before deposition and ALD-CeO_*x*_ of two different thicknesses grown at 160 °C on Si using H_2_O as a coreactant; b) comparison of the corresponding Ce^3+^-cation concentration evolution derived from the O 1s and Ce 3d core levels, depending on the number of ALD cycles.

In addition to the previously discussed coreactant dependence, further investigations of the cation oxidation state in 2.4 nm (15 ALD cycles) thin films reveal a strong dependence of the Ce^3+^ fraction on the choice of substrate (Figure 6). In this series of experiments, we used O_3_, the oxidizing coreactant that produces the fewest oxygen vacancies in thick films, to isolate the role of the substrate. Switching from a Si substrate covered with native oxide to 300 nm of thermally grown SiO_2_ drastically reduces the Ce^3+^ content in the film to 15–20%. In contrast, an Al_2_O_3_ substrate prepared by ALD yields a Ce^3+^ concentration of around 30%. These differences are likely due to variations in the formation of the interface species. This suggests a higher tendency for silicate formation in native silicon oxide than in thicker thermal SiO_2_, and a potential promotion of aluminates on the ALD-Al_2_O_3_ substrate, both of which would pin the Ce cation oxidation state at +3.

**Figure 6:**
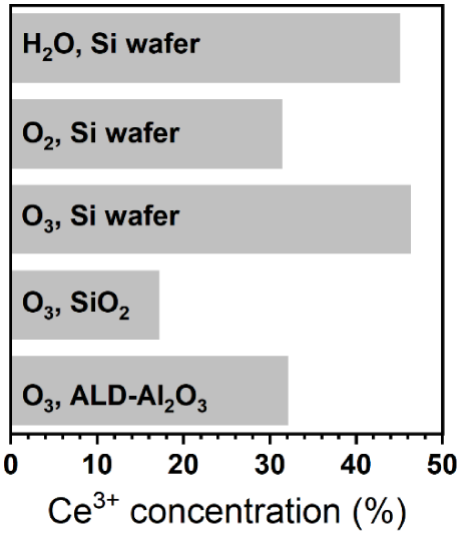
Ce^3+^ concentration of 2.4 nm thick ALD-CeO_x_ (15 ALD cycles) grown using different coreactants and substrates.

This section highlights the importance of understanding how various factors, including the oxygen source, film thickness, morphology, and substrate, influence the cation oxidation state of the ALD-CeO_*x*_ ultrathin layers. We have demonstrated that significant changes in Ce^3+^ concentration can occur even when ALD growth settings, such as temperature and substrate, are held constant. For example, the total fraction of Ce^3+^ species varies between approximately 10% and 30% for thick cerium oxide films grown on silicon, depending on the coreactant. The ability to control the Ce^3+^/Ce^4+^ ratio enables tunability of the cation oxidation state of CeO_*x*_ for different applications by suitably combining deposition parameters, including the substrate, oxygen source, number of ALD cycles, and substrate temperature. Similarly, the differences in surface coverage observed between the different coreactants may be exploited to tune the size of ceria nanoislands for surface functionalization, especially relevant to applications involving gas/surface interactions, such as gas sensing and catalysis.^27^

### Evolution of Precursor-Related Contamination

In an ideal ALD process, the ligands of the adsorbed organometallic molecule are entirely removed from the film during surface reactions with the coreactant; however, precursor remnants can still be frequently found in ALD deposits. These chemical defects crucially influence material properties such as carrier trap density and electrical resistivity, as shown for other metal oxides prepared by ALD.^77,78^ Therefore, it is crucial to investigate the presence of potential residues, the influence of cycle number and coreactant, and how these defects may impact physicochemical properties relevant to future applications.

From the chemical formula of the dpdmg ligand (N_3_C_9_H_20_), the obvious candidates for potential precursor residues are N- and C-containing species. Starting with potential N residues, Figure 7b illustrates the evolution of the N/Ce fraction for cerium oxide grown by ALD using H_2_O, O_2_, and O_3_, respectively, depending on the ALD cycle number. When using H_2_O, the N/Ce ratio is low and independent of the number of ALD cycles, indicating a minor presence of N-impurities in the film. The N 1s spectrum (Figure 7a) reveals a single peak centered at ∼398–400 eV (gray rectangle), which indicates the presence of organic N-compounds related to incomplete removal of some dpdmg ligands during the oxidation half-cycle, similar to well-established nitrogen-containing metal–organic precursors, such as TDMASn^78^ or TDMAHf.^79^

**Figure 7:**
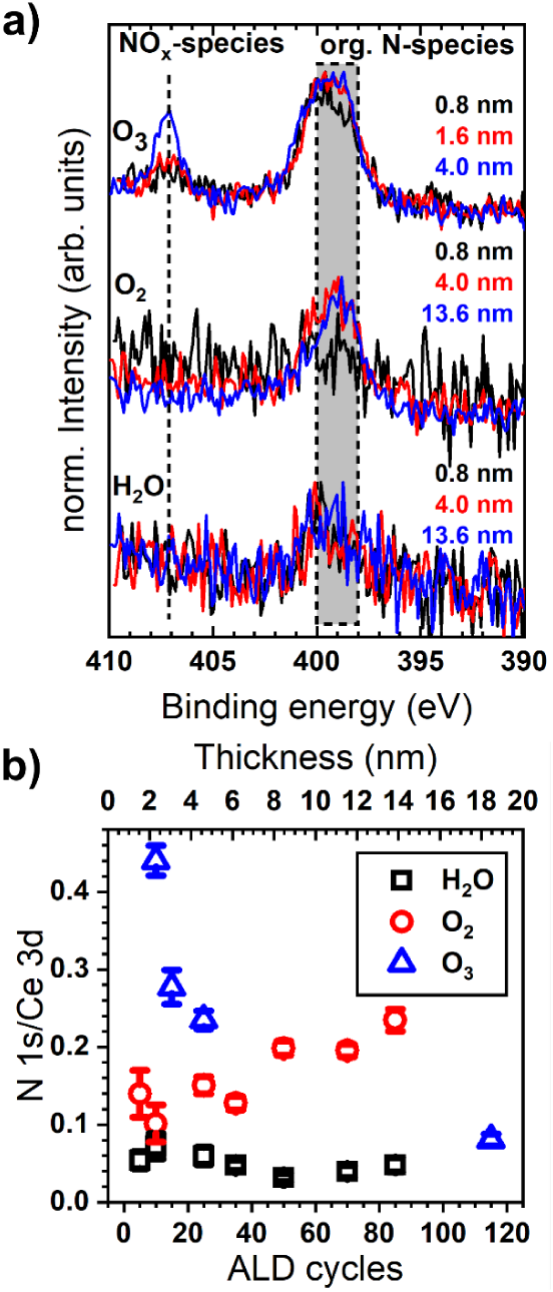
a) N 1s spectra of ALD-CeO_*x*_ grown with [Ce­(dpdmg)_3_] and H_2_O (bottom), O_2_ (middle), and O_3_ (top). b) Evolution of the N 1s/Ce 3d ratio depending on ALD cycle number for CeO_*x*_ grown at 160 °C on Si using H_2_O (black squares), O_2_ (red circles), or O_3_ (blue triangles), respectively. The gray rectangle and black dashed line indicate the binding energy ranges mentioned in the text.

Films grown with O_3_ exhibit an entirely different evolution of the N/Ce ratio. For ultrathin films, the N content is significantly higher than that observed for H_2_O, followed by an exponential decrease to the same ratio as for films grown with H_2_O. This evolution may be explained by N-species located at the surface. Assuming that the surface area does not vary significantly, yielding a near-constant intensity associated with N-species, the Ce 3d intensity would increase following the equation $I\left(d\right)=I_{0}^{\infty }\cdot \left(1-\exp\left(-d/\lambda \right)\right)$ , where $I_{0}^{\infty }$ is the intensity of an infinitely thick ceria layer, d is its thickness, and λ is the attenuation length (almost identical to IMFP), yielding an exponential decrease of the N/Ce ratio toward a constant value. The nature of the N-species also evolves with the number of ALD cycles. While initially only a single component related to organic N-compounds appears at 398–400 eV, a second peak arises around 407 eV at a film thickness of 4 nm (25 ALD cycles), which is consistent with adsorbed NO_*x*_-species^80^ occupying surface sites or the formation of NO_3_-impurities with similar binding energy as other rare-earth nitrates.^81^ The comparison between H_2_O and O_3_ in the heterodeposition regime provides valuable insights into potential changes in the reaction mechanisms, which seem to transition from the removal of complete ligands (or large parts) in the case of H_2_O, to a combustion mechanism in the case of O_3_, leading to the formation of H_2_O, CO_2_, and NO_*x*_ species. Moreover, thick CeO_*x*_ films grown with O_3_ exhibit an N 1s spectrum with a single feature at 398–400 eV (Figure S5 in Supporting Information). A potential explanation for this observation is the presence of certain N-species at the substrate/ceria interface, which gives rise to the peak at 407 eV. As surface coverage increases during the initial stages of deposition, the intensity of this component increases, resulting in a clear peak at 407 eV after 25 cycles rather than the broad feature observed for fewer ALD cycles. Once the interface is buried beneath a thick layer and the growth enters the homodeposition stage, in which the reaction mechanism may change, the XPS probing depth is too shallow to detect the interface species. Therefore, strongly oxidized or organic N-species are found at the interface or at the very top surface when using O_3_.

Finally, films grown with O_2_ exhibit a peculiar behavior in the N/Ce fraction, as it progressively increases with cycle number. For comparison, the atomic percentages of nitrogen are 6.2 at%, 1.3 at%, and 2.4 at% for samples grown with O_2_ (13.6 nm thickness), H_2_O (13.6 nm thickness), and O_3_ (18.4 nm), respectively. While no definitive explanation can be derived solely from the XPS measurements, the presence of a single peak in the N 1s spectrum at 400 eV indicates the accumulation of organic species, likely arising from the incomplete removal of precursor ligands during surface reactions. This may be corroborated by DFT calculations presented by Kaur et al.,^50^ showing that the reaction of [Ce­(dpdmg)_3_] with H_2_O results in the formation of a Ce–O bond concomitant with the cleavage of a Ce–N bond, while the reaction between [Ce­(dpdmg)_3_] and O_2_ yields a Ce–O bond and the insertion of an oxygen atom into the Ce–N bond, resulting in a Ce–O–N bond. This may indicate that oxygen is not an ideal coreactant; however, further investigations, e.g., by Fourier-transform infrared spectroscopy, are needed to confirm this hypothesis.

In addition to nitrogen impurities, carbon impurities have been investigated for cerium oxide grown with H_2_O, O_2_, and O_3_, as shown in Figure 8. To extract information on the chemical environment, a simple model based on the literature^56^ has been applied, using the following components: C–C/C–H bonds, C–O bonds (+1.5 eV), O–C–O/C=O bonds (+3 eV), and O–C=O-bonds (+4 eV), together with a peak compensating for the Ce 4s core level, which is also found in this region. Additional carbon-containing species, such as those with C–N bonds, may also be present at lower concentrations. However, their binding energy is relatively close to that of other components included in the fit, e.g., C–O and C–N species, which would lead to high correlation between the different components, as they cannot be resolved with a nonmonochromatized X-ray source. Therefore, their inclusion would significantly complicate the analysis and yield only minimal additional insights. Exemplary C 1s spectra and their respective fits of ALD-CeO_*x*_ grown with H_2_O as the oxidizing agent are displayed in Figure 8a, while the evolution of the different fit components of the C 1s model is shown in Figure 8b–d for CeO_*x*_ grown with H_2_O, O_2_, and O_3_, respectively.

**Figure 8:**
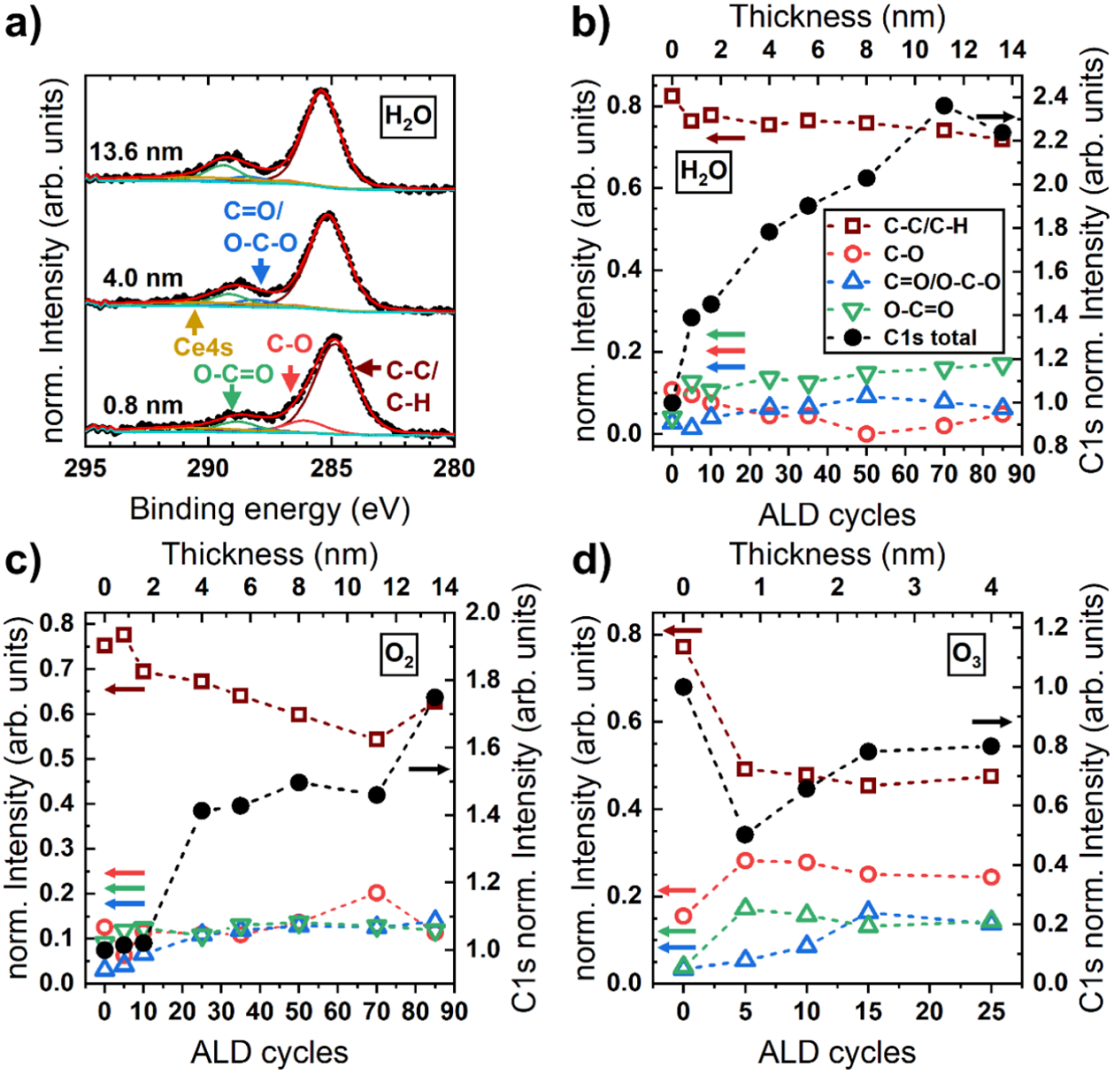
a) Evolution of C 1s spectra of ALD-CeO_*x*_ grown with [Ce­(dpdmg)_3_] and H_2_O. b) Evolution of the total C 1s intensity (normalized to bare substrate) and the relative amount of different C species derived from fits of the C 1s spectra of ALD-CeO_*x*_ grown with b) H_2_O, c) O_2_, and d) O_3_, respectively.

An easily accessible quantity derived from the C 1s spectra is the evolution of the total intensity with an increasing ALD cycle number. As the sample cools in the ALD reactor after deposition, some organic species may adsorb on the surface before the sample is transferred to the analysis chamber, leading to unavoidable *adventitious* carbon. The intensity of these species is directly related to the sample surface area, which may change during film deposition, especially during the initial cycles when the total coverage of the nanoislands increases. Following this reasoning, an increased C 1s intensity in thick films would indicate additional C species located within the film, and vice versa.

When using H_2_O and O_2_ as coreactants, the total amount of carbon measured by XPS increases by factors of 2.2 and 1.8, respectively, for film thicknesses of 13.6 nm (compared to the initial amount). This indicates the presence of carbon within the film in addition to some accumulation on the sample surface, as discussed in the previous paragraph. For H_2_O, the different carbon species derived from the C 1s fit seem to exhibit constant proportions with ALD cycle number, dominating the C–C/C–H component. The latter is also true for films grown with O_2_, although the C–C/C–H fraction is continuously decreasing from ∼75% for 0.8 nm ALD-CeO_*x*_ films to ∼60% (13.6 nm film thickness), concomitant with a minor increase in the other carbon-bonded-to-oxygen components. In any case, the carbon content does not saturate when O_2_ is used, as with the N-species, again indicating the inclusion and accumulation of organic species.

In contrast, the samples grown with O_3_ show an overall decrease in C 1s intensity with increasing film thickness, accompanied by a decrease in the C–C/C–H fraction to values similar to those for cerium oxide grown with O_2_. These findings suggest that coreactants with higher oxidation potential are more effective at preventing C incorporation into the film. For samples with a thickness of 13.6 nm, grown with H_2_O and O_2_, the atomic percentage of carbon is 25 at% and 24.6 at%, while an 18.4 nm sample grown with O_3_ exhibits the lowest carbon concentration of 12.8 at%. These values are likely overestimates because a significant portion of carbon is located on the surface. The substantial difference in overall C 1s intensity between ALD growth using H_2_O and O_3_ may also be related to a change in reaction mechanism, from removal of the entire remaining ligands to their combustion. Whereas larger organic molecules, as a result of the ligand removal (H_2_O case), may remain in the chamber for longer and adsorb onto the sample during sample cooling (*adventitious* contribution in C 1s), smaller molecules (O_3_ case), resulting from ligand combustion (H_2_O, CO_2_, and NO_*x*_) are evacuated from the chamber more readily.

Summarizing this section, C and N impurities exhibit distinct dependencies on film thickness and coreactant. While a deeper understanding of the mechanism requires further investigation of reaction products and intermediates during the half-cycles, the discussion of potential reaction pathways offers additional insights. ALD involving H_2_O as the coreactant generally assumes a ligand-exchange mechanism between adsorbed precursor species and H_2_O. With -| denoting the species adsorbed on the surface, the following reactions would be expected for the [Ce­(dpdmg)_3_] precursor:

1 $$\left[\mathrm{Ce}{\left(\mathrm{dpdmg}\right)}_{3}\right]+\mathrm{x OH}-\left|\to \left[\mathrm{Ce}{\left(\mathrm{dpdmg}\right)}_{3-x}\right]-O_{x}\;-\;\right|+{\mathrm{x Hdpdmg}}_{\left(g\right)}$$

2 $$\left[\mathrm{Ce}{\left(\mathrm{dpdmg}\right)}_{3-x}\right]-O_{x}-\left|+\left(3-x\right)H_{2}O\to {\left(\mathrm{HO}\right)}_{3-x}-\mathrm{Ce}-O_{x}\;-\;\right|+\left(3-x\right){\mathrm{Hdpdmg}}_{\left(g\right)}$$

Although this mechanism would lead to the formation of pure Ce_2_O_3_, oxidation to (sub)­stoichiometric CeO_2‑x_ can be expected even under mild conditions. Including the potential carbodiimide-deinsertion mechanism hypothesized earlier, the reaction product would be $\mathrm{HN}{\left({\mathrm{CH}}_{3}\right)}_{2}$ instead of Hdpdmg during both half-cycles. These simple considerations suggest the presence of large, organic molecules that may readsorb on the surface or remain behind due to incomplete reactions, offering a potential explanation for the high fraction of C–C/C–H species in the C 1s fits and the presence of organic N species in the N 1s core level.

When O_3_ is used as the coreactant, the adsorption process still follows reaction (1), while the remaining ligands of the precursor adsorbates combust to form small molecules, such as H_2_O, CO_2_, NO_*x*_, etc. Gas-surface interactions of H_2_O lead to surface hydroxylation for the following precursor dose, and the elevated temperature facilitates the evacuation of gas-phase species from the ALD reactor. If the Ce–N bond cleavage is not energetically favorable, some Ce–N–O_*x*_ species may form during ligand combustion, which may explain the N 1s signal observed at ∼407–408 eV in some measurements.

ALD with O_2_ may proceed via an intermediate reaction mechanism: DFT calculations by Kaur et al.^50^ indicate that one oxygen atom inserts into the Ce–N bond, whereas the reaction with H_2_O yields a Ce–OH bond and an N–H bond. The resulting Ce–O–N bond may hinder the efficient removal of N species, providing a potential explanation for the observed increase in the Ce/N ratio. Continuous accumulation of N in CeO_*x*_ films grown with O_2_ may pose a significant obstacle to the use of this precursor-*co*-reactant combination in subsequent studies.

The observation that N impurities are effectively suppressed when H_2_O is used, whereas the lowest C incorporation occurs with O_3_, suggests that other oxygen sources, such as H_2_O_2_, may help minimize C and N impurities simultaneously by providing oxygen and hydrogen for efficient carbon and nitrogen removal while increasing reactivity compared to H_2_O. Additionally, increasing the deposition temperature above 160 °C or using plasma-activated species as the oxygen source could be viable strategies to reduce the risk of incomplete precursor-*co*-reactant reactions.

Moreover, controlling the incorporation of carbon or nitrogen may be a suitable route for tuning material properties such as conductivity, as reported by Chistiakova et al.^78^ for SnO_*x*_ and highly relevant for conductometric gas sensing, or for tuning the band gap and the presence of in-gap states for applications such as (photo)­catalysis, as reviewed by Marschall and Wang for TiO_2_.^82^

## Conclusions

Precise control over chemical and structural defects is vital for future applications of ultrathin metal oxide films. Although significant efforts have traditionally focused on minimizing defect density via physical or chemical vapor deposition approaches, ALD offers additional opportunities to tailor the type and concentration of intrinsic defects in ultrathin films. Such defect engineering can, in turn, affect material properties for applications such as gas sensing and heterogeneous catalysis by modifying existing active sites or promoting new ones.

Here, we demonstrated the potential of thermal ALD to precisely engineer defects in ultrathin CeO_*x*_ layers by tuning key deposition parameters. Employing *in situ* XPS, crucial insights into the role of coreactants during CeO_*x*_ growth with the [Ce­(dpdmg)_3_] precursor were obtained, enabling the identification of strategies to tune the oxygen vacancy concentration in the film. The ALD process was performed at relatively low temperatures of 160 °C, with linear deposition observed down to 140 °C—the lowest value reported to date for thermal CeO_*x*_ ALD. While the growth rate is coreactant-independent, initial film coverage depends strongly on the chosen oxygen source, with the fastest and slowest layer closures observed for H_2_O and O_3_, respectively. Oxygen vacancy concentration, derived from the Ce 3d core level, can be tuned through coreactant, thickness, and substrate choice. Together, these findings open a promising avenue for simultaneously adjusting nanoisland size and vacancy concentration. The distinct coreactant dependence of carbon and nitrogen impurities highlights a shift in the precursor-*co*-reactant reaction mechanism. These results showcase the suitability of ALD-based cerium oxide for a wide range of applications, including catalysis and gas sensing.

Furthermore, our investigations provided insights into the associated ALD reaction mechanism. Interestingly, theoretical GPC estimates based on the adsorbate size of [Ce­(dpdmg)_*x*_] species and assuming a pure ligand-exchange mechanism cannot account for the experimentally observed values. This suggests a more complex adsorption scenario beyond the loss of two ligands, such as partial decomposition of the remaining dpdmg ligand or carbodiimide deinsertion, which could significantly influence the formation and concentration of chemical defects. This behavior is likely not unique to the [Ce­(dpdmg)_3_] precursor and could also be relevant in other ALD processes employing metal precursors with amidinate or guanidinate ligands that exhibit high GPC values.

## Supplementary Material



## Notes

Table of fit constraints used for XPS Ce 3d fit; ellipsometry data of thermal ALD-CeO_*x*_ process using O_2_ and O_3_ as coreactants, respectively; O 1s fit of CeO_*x*_ grown with O_2_ or O_3_, respectively, and comparison to Ce^3+^ concentration derived from Ce 3d core level analysis; N 1s spectrum of CeO_*x*_ grown with O_3_ after 115 ALD cycles (PDF)

## Abbreviations

ALD: Atomic layer deposition
GPC: Growth per cycle
IMFP: Inelastic mean free path
IPSA: Inelastic peak shape analysis
PVD: Physical vapor deposition
VOC: Volatile organic compound
XPS: X-ray photoelectron spectroscopy
XRR: X-ray reflectivity
