# Supplementary material for: Defect Engineering in Atomic-Layer-Deposited Cerium Oxide
Source: ACS Appl Mater Interfaces. 2026 Mar 16;18(12):18380–93. doi: 10.1021/acsami.5c22734 (PMC13051452; doi:10.1021/acsami.5c22734)
Supplement: Supplementary file 1 [file am5c22734_si_001.pdf]

Supporting Information

Defect Engineering in atomic-layer deposited cerium oxide

Rudi Tschammer[1], Marcel Schmickler[2,4], Yuliia Kosto[1,3], Karsten Henkel[1], Parmish Kaur[4], Anjana Devi[2,5,6], Carlos Morales[1]*, and Jan Ingo Flege[1]*

[1] Applied Physics and Semiconductor Spectroscopy, BTU Cottbus-Senftenberg, 03046 Cottbus, Germany

[2] Leibniz Institute for Solid State and Materials Research, 01069 Dresden, Germany

[3] Faculty of Mathematics and Physics, Department of Surface and Plasma Science, V Holešovičkách 2, Charles University, 180 00 Prague 8, Czech Republic

[4]Inorganic Materials Chemistry, Ruhr University Bochum, 44801 Bochum, Germany

[5]Chair of Materials Chemistry, TU Dresden, 01069 Dresden, Germany

[6]Fraunhofer Institute for Microelectronic Circuits and Systems (IMS), 47057 Duisburg, Germany

*Corresponding Authors: Jan Ingo Flege Email: flege@b-tu.de

Carlos Morales Email: moraless@b-tu.de

	Ce^{4+}						Ce^{3+}			
Peak	**v**	**v''**	**v'''**	**u**	**u''**	**u'''**	**v0**	**v'**	**u0**	**u'**
Line shape	SGL(40) T(1.25)	SGL(40) T(1.1)	SGL(50)	SGL(45) T(1.25)	SGL(40) T(1.1)	SGL(40)	SGL(20)	SGL(40)	SGL(20)	SGL(40)
Area		$v \cdot 0.8$	$v \cdot 0.95$	$v \cdot {}^{2}/_{3}$	$v'' \cdot {}^{2}/_{3}$	$v''' \cdot {}^{2}/_{3}$		$v0 \cdot 1.8$	$v0 \cdot {}^{2}/_{3}$	$v' \cdot {}^{2}/_{3}$
FWHM (eV)		$v \cdot 1.8$	$v \cdot 1.14$	$v \cdot 0.86$	$v \cdot 1.8$	$v \cdot 1.1$		$v0 \cdot 1.2$	$v0 \cdot 1$	$v0 \cdot 1.2$
Position (eV)	$u''' - 34.3$	$u''' - 28.2$	$u''' - 18.4$	$u''' - 15.8$	$u''' - 9.4$	917.0	$\sim u''' - 35.8$	$v0 + 4.4$	$v0 + 18.5$	$v0 + 22.9$

Table S1. Overview of fit constraints applied to the sub-peaks corresponding to Ce^{4+} and Ce^{3+} oxidation states during the fit of the Ce 3d core level.

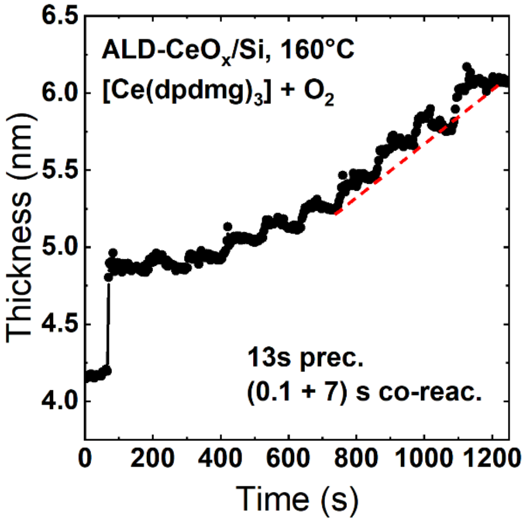

Figure S1. Time-dependent thickness evolution determined by spectroscopic ellipsometry for a cerium oxide film grown via thermal ALD cycles at 160 °C composed of a 13 s [Ce(dpdmg)$_3$] pulse and a 0.1 s O_2 pulse, followed by 7 s of pumping before the purge step. The initial step and the lower GPC observed during the first five cycles are always observed when the ALD process is started, regardless of the sample surface or selected co-reactant. The red line indicates the cycles when the thickness evolution has recovered to a steady, linear increase.

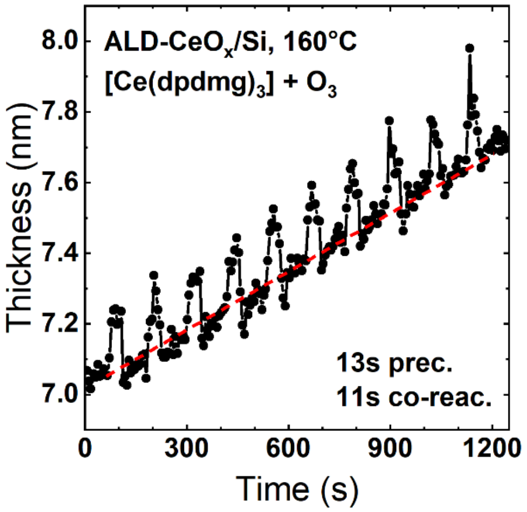

Figure S2. Time-dependent thickness evolution determined by spectroscopic ellipsometry for a cerium oxide film grown via thermal ALD cycles at 160 °C composed of a 13 s [Ce(dpdmg)$_3$] pulse and a 11 s O_3 pulse. Red dashed lines have been added to guide the reader's eye.

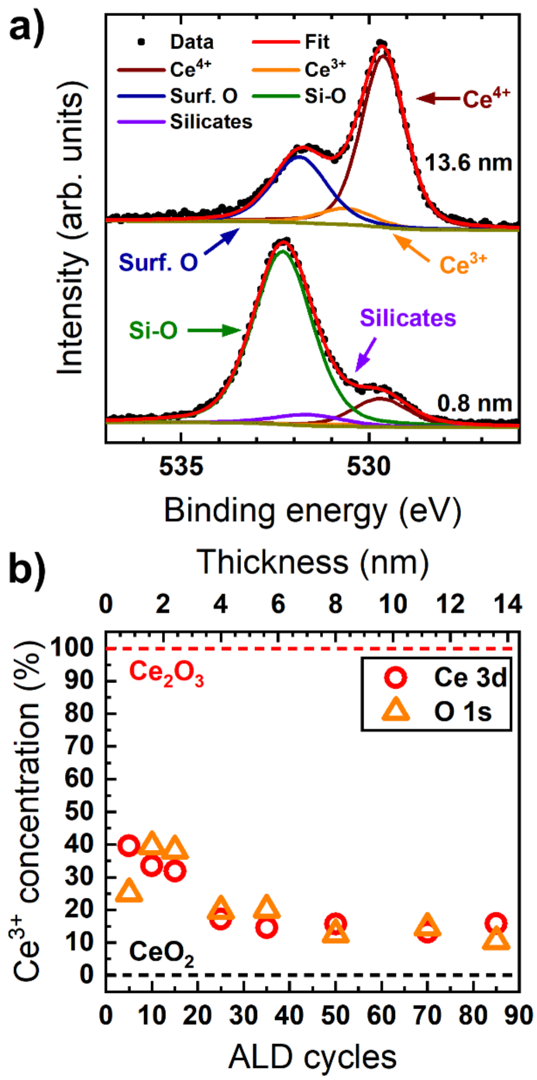

Figure S3. a) Comparison of O 1s spectra for two different thicknesses of ALD-CeO_x deposits grown at 160 °C on Si using O_2 as a co-reactant; b) comparison of the corresponding Ce^{3+}-cation concentration evolution derived from the O 1s and Ce 3d core levels, depending on the number of ALD cycles.

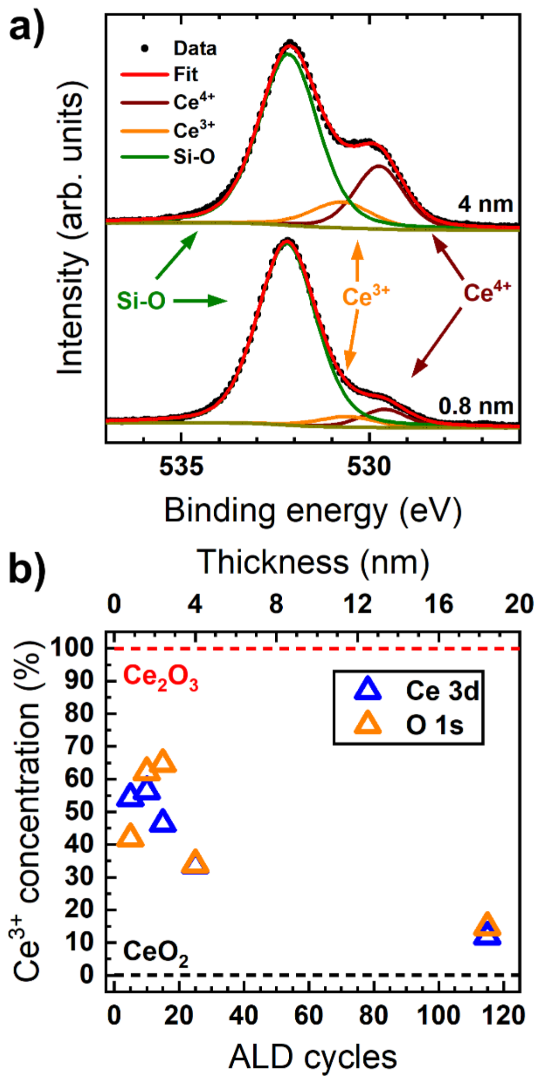

Figure S4. a) Comparison of O 1s spectra of ALD-CeO_x deposits of two different thicknesses grown at 160 °C on Si using O_3 as a co-reactant; b) comparison of the corresponding Ce^{3+}-cation concentration evolution derived from the O 1s and Ce 3d core levels, depending on the number of ALD cycles.

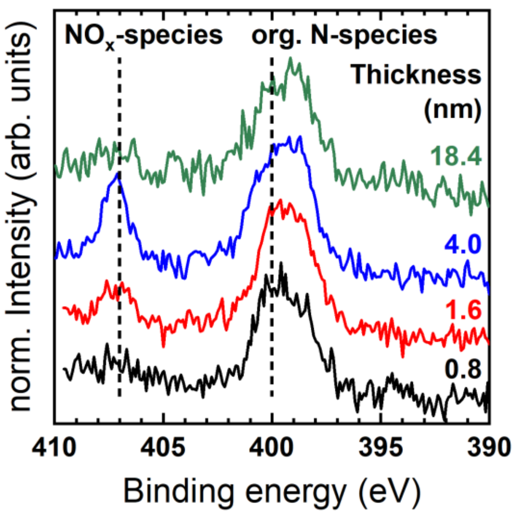

Figure S5. Evolution of the N 1s core level depending on the number of ALD cycles of CeO_x grown at 160°C on a Si substrate using [Ce(dpdmg)$_3$] and O_3.
